# An Analysis of Patient-Reported Outcomes Measurement Information System (PROMIS) in Non-operative Posterolateral Elbow Dislocations

**DOI:** 10.7759/cureus.43297

**Published:** 2023-08-10

**Authors:** Thomas J Carroll, Akhil Dondapati, Jonathan Minto, Samantha Hoffman, Warren C Hammert, Bilal Mahmood

**Affiliations:** 1 Department of Orthopaedic Surgery, University of Rochester, Rochester, USA; 2 Department of Orthopaedic Surgery, Division of Hand Surgery, Duke University Medical Center, Durham, USA

**Keywords:** upper extremity trauma, promis scores, elbow dislocation without fracture, traumatic elbow dislocation, simple posterior elbow dislocation

## Abstract

Introduction: The purpose of our study is to analyze the outcomes of traumatic posterolateral elbow dislocations using patient-reported outcomes measurement information system (PROMIS). We hypothesized that physical function (PF) and upper extremity (UE) scores in PROMIS will significantly improve over six months of follow-up and correlate with a positive change in the patient-acceptable symptom state (PASS).

Methods: This is a seven-year retrospective study of 165 consecutive adult patients with traumatic posterolateral elbow dislocations. Demographic information, PROMIS PF, PROMIS UE, PROMIS pain interference (PI), PROMIS depression, and PASS were recorded over six months of follow-up.

Results: At the time of injury, mean PROMIS scores were PF 41.24 (SD 11.16), UE 34.27 (SD 11.87), PI 60.44 (SD 8.07), and depression 49.82 (SD 10.42). At six months, the mean PROMIS scores were PF 39.71 (SD 9.71), UE 33.95 (SD 9.09), PI 57.35 (SD 8.59), and depression 51.43 (SD 10.62). The overall six-month changes in PROMIS scores were PF -1.53, UE -0.32, PI -3.09, and depression +1.61. At the 6-month follow-up, 41.7% responded positively on the PASS, which correlated only with PROMIS PI.

Conclusions: Among patients who improved from negative to positive response on PASS, the PROMIS PF, UE, and depression scores did not significantly improve. Only PROMIS PI correlated with PASS at the six-month follow-up; PROMIS PI significantly improved among simple posterolateral elbow dislocation patients at both short-term and long-term follow-up points. PROMIS PF, UE, and depression did not significantly differ between time of injury and short-term and long-term follow-up points.

## Introduction

The elbow joint is the second most commonly dislocated joint in adults, with an incidence of 5.21 per 100,000 person-years [1]. Simple dislocations are those in which there are no concomitant fractures, or only small periarticular avulsions < 2mm in diameter. They occur more frequently in male patients and are usually a result of falls from a standing height [2-7]. The elbow joint itself has substantial bony stability [8,9]. The primary stabilizers of the elbow joint include the medial collateral ligament (MCL), lateral ulnar collateral ligament (LUCL) complex, and ulnohumeral articulation. Muscles traversing the elbow allow for dynamic stabilization [10-12]. Disruption of multiple structures results in dislocation, the most common being a posterior or posterolateral dislocation [2].

Diagnosis of an elbow dislocation involves a detailed history and physical examination of the patient, as well as anteroposterior and lateral radiographs. Treatment for most elbow dislocations is non-operative, with reduction and initial immobilization. After reduction, the elbow should be moved through its full range of motion to determine stability and radiographs should be obtained to confirm reduction [13]. Surgical management for simple dislocations is controversial, with indications mostly depending on joint incongruity and the extent of soft tissue injury. Barring these indications, studies have demonstrated no notable differences in re-dislocation rates or range of motion for non-operative versus operative patients [4]. After an elbow dislocation, it can be expected for some patients to have residual pain and stiffness, but functional outcomes are generally satisfactory. After initial reduction, there is evidence to support that immobilization longer than three weeks is associated with poorer elbow range of motion and function [11,12]

Patient-reported outcomes measurement information system (PROMIS) scores are measured utilizing a normally distributed T-score metric, with a mean of 50 and an SD of 10 in the United States general population. Higher scores for PROMIS physical function (PF) and upper extremity) indicate greater functionality. Conversely, higher scores of PROMIS pain interference (PI) and depression represent greater impairment. Patient-acceptable symptom state (PASS) is one question: Is your current condition acceptable?

The purpose of our study is to analyze the post-injury outcomes of adult patients with traumatic posterolateral elbow dislocations using PROMIS. Our hypothesis is that PROMIS PF, UE, PI, and depression correlate with positive change in the PASS at the six-month follow-up. Our secondary hypothesis is that PROMIS PF, UE, PI, and depression scores would improve over the course of our study, between the time of injury and the six months of follow-up.

## Materials and methods

The study was approved by the University of Rochester Institutional Review Board (IRB) (approval number: MOD00018982). A waiver of consent was granted by the IRB as this was a retrospective evaluation of a prospectively collected database at a single, urban Level 1 trauma center. PROMIS PF (v1.2/2.0), UE (v2.0), PI (v1.1), depression (v1.0), and PASS v1.0 instruments using computer adaptive test (CAT) were collected at routine clinic visits between October 1, 2015, and March 1, 2022, on Apple iPads/tablets (Apple Inc., Cupertino, California, United States).

Patients included in this study were identified utilizing Current Procedural Terminology (CPT®) codes 24600 and 24605, as well as the International Classification of Disease (ICD-10) code S53.X. Charts were reviewed to ensure they were diagnosed with a simple posterolateral elbow dislocation based on history, physical exam, and radiographic confirmation, and underwent closed reduction under sedation. Inclusion criteria were: patients aged 18-75 years, who had a closed reduction between October 2015 and March 2022 at our institution. Additional inclusion criteria included PROMIS data collected during at least one follow-up appointment after treatment and at least six weeks of total follow-up. Patients missing initial post-injury follow-up (within one week of injury) or the six-week follow-up were excluded. Exclusion criteria were ages outside the range of 18-75 years, patients with multiple injuries (i.e. polytrauma), complex elbow dislocations, previous ipsilateral elbow dislocation, history of impaired elbow function at baseline, or initial surgical intervention. Patients who underwent associated surgery at any point during the follow-up period were also excluded. Patients were immobilized for an average of four weeks. The data was de-identified and securely stored within the hospital network. 

Statistical analysis was performed with Microsoft Excel® (Microsoft Corporation, Redmond, Washington, United States) and RStudio (Version 2022.07.0; R Foundation for Statistical Computing, Vienna, Austria). Descriptive statistics including mean, SD, and frequency were calculated for all demographic variables. The SD, mean, and standardized response mean (SRM) were calculated for every PROMIS domain evaluated at each study time point. The paired t-test was used to compare PROMIS means of the same cohort between different time points. Unpaired t-test and chi-squared test were used to compare demographic variables. The Cohen SRM, calculated as the difference in the pre-treatment and post-treatment means divided by the SD of the difference, is an effect size index used to gauge an instrument’s responsiveness, which is defined as an instrument’s sensitivity to change over time [14]. Using the definition of Cohen’s effect size, 0.2-0.49 was considered a small response, 0.5-0.79 was a moderate response, and 0.8 or greater was a large response. Values of p < 0.05 were considered statistically significant. Calculations for PROMIS/PASS analysis at six weeks and six months included only patients with both scores completed.

## Results

We identified 165 patients who underwent closed reduction of a posterolateral elbow dislocation and who met the criteria for inclusion. Descriptive characteristics of the included patients are reported in Table 1. Across all patients, the average age was 25 years (SD: 11 years), 57% of patients were female (n = 94), and the average BMI was 29 kg/m^2^ (SD: 5) (Table 1).

**Table 1:** Baseline Characteristics of All Patients with Simple Posterolateral Elbow Dislocation Undergoing Closed Reduction

Characteristic	n (%) or mean (SD)
Age, years	25 (11)
BMI	29 (5)
Sex	
Female	94 (57%)
Male	71 (43%)
Race	
White	142 (86%)
Black	21 (13%)
Other	2 (<1%)
Ethnicity	
Not Hispanic	155 (94%)
Hispanic	2 (<1%)
Unknown	8 (5%)
Affected Side	
Left	45 (27%)
Right	120 (73%)

At the initial outpatient post-injury visit (within one week of injury), the average PROMIS PF, UE, PI, and depression were 41.24 (SD: 6.8), 34.27 (SD: 7.8), and 60.44 (SD: 7.9), and 49.82 (SD: 10.2), respectively. At this time, among the patients who completed the PASS question, 15 (18.3%) reported an acceptable symptom state. At the six-week post-injury visit, the average PROMIS PF, UE, PI, and depression scores were 42.07 (SD: 5.8), 36.39 (SD: 8.2), 57.12 (SD: 6.4), and 47.70 (SD:11.4), respectively. At the six-month post-injury visit, the average scores were 39.71 (SD: 7.2), 33.95 (SD: 8.3), 57.35 (SD: 5.9), and 51.43 (SD: 9.8), respectively, amongst the 68 patients with completed data. 

The average changes in PROMIS PF, UE, PI, and depression scores from the time of injury to six months were -1.53 (p=0.1), -0.32 (p=0.7), -3.09 (p<0.001), and 1.61 (p=0.08), respectively. The complete PROMIS data at all follow-up time points are summarized in Table 2. PROMIS PI demonstrated statistically significant improvement at both the six-week and six-month follow-up (p<0.001). PROMIS PF, UE, and depression were not significantly different between the initial visit and all follow-up time points. The standard response means for PROMIS PF, UE, PI, and depression at six months were -0.22, -0.04, -0.45, and 0.15, respectively, which are classified as small responses (Table 3).

**Table 2:** PROMIS Physical Function, Upper Extremity, Pain Interference, And Depression Scores at Different Follow-Up Points PROMIS: Patient-Reported Outcomes Measurement Information System; PF: Physical Function; UE: Upper Extremity; PI: Pain Interference

Dislocation	PROMIS PF	PROMIS UE	PROMIS PI	PROMIS Depression
Time of Injury	41.24	34.27	60.44	49.82
2 Weeks	40.72	38.83	57.82	47.23
6 Weeks	42.07	36.39	57.12	47.70
12 Weeks	41.98	37.05	57.52	48.34
6 Months	39.71	33.95	57.35	51.43
Change (6 months – Initial)	-1.53	-0.32	-3.09	1.61
	p=0.1	p=0.7	p<0.001	p=0.08

**Table 3:** Results from PROMIS Upper Extremity, Physical Function, Pain Interference, and Depression with Six-Week and Six-Month Standard Response Mean PROMIS: Patient-Reported Outcomes Measurement Information System; SRM: Standard Response Mean

Outcome Measure	Initial Visit, mean (SD)	6 Weeks, mean (SD)	6 Weeks, SRM	6 Months, mean (SD)	6 Months, SRM
PROMIS Physical Function	41.24 (6.8)	42.07 (5.8)	0.13	39.71 (7.2)	-0.22
PROMIS Upper Extremity	34.27 (7.8)	36.39 (8.2)	0.27	33.95 (8.3)	-0.04
PROMIS Pain Interference	60.44 (7.9)	57.12 (6.4)	-0.47	57.35 (5.9)	-0.45
PROMIS Depression	49.82 (10.2)	47.70 (11.4)	-0.19	51.43 (9.8)	0.15

At the six-month visit among the patients who completed the PASS question, 41% reported an acceptable symptom state. Among patients initially reporting “not acceptable” on PASS and reporting “acceptable” at the six-month visit, the average PROMIS PF, UE, PI, and depression scores were 43.21, 34.94, 56.43, and 50.98, respectively. This represents an average difference of 1.19 (p=0.19), 0.93 (p=0.3), -4.09 (p<0.01), and -2.23 (p=0.06), respectively. These results reflect patients with both six-month PROMIS and PASS questionnaires completed. PROMIS PI demonstrated a clinically appreciable improvement when considering minimal clinically important differences (MCID) estimates using 1/3 standard deviation but not 1/2 standard deviation (six-month MCID, 3.4) [15]. Neither PROMIS PF, UE, nor depression demonstrated a clinically significant improvement at any time point. PROMIS UE scores worsened slightly at six months for those responding “acceptable” to the initial PASS question. PROMIS PF scores improved, but neither changes were statistically significant. The complete PASS versus PROMIS data can be found in Table 4.

**Table 4:** PROMIS Upper Extremity, Physical Function, Pain Interference, Depression Among Patients Completing The PASS PROMIS: Patient-Reported Outcomes Measurement Information System; PF: Physical Function; UE: Upper Extremity; PI: Pain Interference; PASS: Patient-Acceptable Symptom State

	Initial Visit				6-Month Visit (difference)			
PASS Question	PROMIS PF	PROMIS UE	PROMIS PI	PROMIS Depression	PROMIS PF	PROMIS UE	PROMIS PI	PROMIS Depression
PASS: Acceptable	40.09	35.21	60.34	50.01	43.21 (3.12)	34.94 (-0.27)	56.43 (-3.91)	50.98 (0.98)
PASS: Not Acceptable	42.01	34.01	60.52	53.21	38.24 (-3.77)	33.62 (-0.39)	58.12 (-2.4)	52.83 (-0.38)
PASS Acceptable – Not Acceptable	-1.92 (p<0.01)	1.2 (p=0.07)	-0.18 (p=0.7)	-3.2 (p<0.001)	4.97 (p<0.001)	1.32 (p=0.06)	-1.67 (p=0.04)	-1.85 (p=0.02)

## Discussion

Similar short-term and long-term patient-reported outcome studies for posterolateral dislocations have reported favorable long-term outcomes. However, they have also noted considerable rates of residual pain and elbow stiffness without functional instability [16]. Historic patient-reported outcomes used in the evaluation of elbow trauma include the Disabilities of the Arm, Shoulder, and Hand (DASH), Short Form-36 Health Survey (SF-36) the Oxford elbow questionnaire, and patient satisfaction questionnaires. Among these tools, DASH and the Oxford elbow score were found to be correlated with objective physical exam measurements of improvement, including range-of-motion and strength after long-term follow-up (mean 88 months) [16-18]

PROMIS has been well established in the evaluation of other extremity conditions, including carpal tunnel syndrome, distal biceps tendon repair, elbow ulnar collateral ligament reconstruction, and elbow arthroscopy [19]. PROMIS PF and UE, specifically, have been shown to be correlated with SF-36 and DASH scores. It was noted, however, that PROMUS UE had a notable ceiling effect in younger, higher-functioning patients, who comprise the majority of this study population [19].

Given that only PROMIS PI showed a significant difference at short-term or intermediate-term follow-up, it is possible that PROMIS PF, UE, and depression are not sensitive enough to detect an improvement for non-operative management of elbow dislocations, despite the success with other conditions. This could be due to the aforementioned ceiling effect among young healthy patients for PROMIS UE or possibly because it does not ask questions that assess recovery and improvement of function for this condition. This shortcoming of PROMIS has been previously noted and may be highlighted in a traditionally non-surgical injury where patients are seen sometimes weeks after the injury [20,21]. This disparity might also be due to a clinically important factor that is not being captured by the PROMIS metrics, including factors such as sleep interference.

An interesting observation is the fact that 18.3% of patients reported an acceptable symptom state on PASS at their initial visit within one week of injury. This might suggest that patients felt subjectively better once they got to the clinic after their elbow was reduced and viewed that closed treatment as a success. A high positive response on PASS at the initial visit might correspond to better baseline PROMIS scores. This might cause a ceiling effect and explain why there is no significant improvement in PROMIS scores over time. One way to address this would be to have pre-injury PROMIS data. This was largely not available within our cohort.

Our study has several notable limitations. Due to the retrospective nature of this project and the relatively variable questionnaire response rate, PROMIS and PASS data were unavailable at some post-injury time points. There was a considerable decrease in questionnaire response rate between initial and final visits with only 41.2% completing follow-up PROMIS scores. We hypothesize that patients who have returned to their baseline or an acceptable level of function may not continue to follow up in the clinic. Some of these patients may also have followed up elsewhere which we would not have detected. This may cause a selection bias in that those who continued to follow up tended to have worse outcomes. This bias therefore may reduce the generalizability of these results to the cohort as a whole.

Lead time bias may also have affected our conclusions. Patients were routinely seen in the clinic up to one week after the initial injury. Over this period, patients may have already improved significantly on PASS/PROMIS. This would reduce the potential improvement over the follow-up duration. Due to the retrospective nature of our study, routine clinical visits and exams closer to the time of injury could not be controlled. 

Additionally, our patients were from an urban level I trauma center in the Northeast United States and might not be reflective of other geographic locations. Further, our cohort was predominantly female in spite of elbow dislocations being more common in males. PROMIS investigators have noted that gender may potentially confound PROMIS scores, which could further alter our findings [22]. We have evaluated the subjective outcomes based on PROMIS and PASS but have not compared them to other legacy patient-reported outcomes or objective physical exam measurements, which may provide additional insight. 

## Conclusions

In this study, we found that only PROMIS PI correlated with PASS at the six-week and six-month follow-ups. Among patients who improved from a negative to a positive response on PASS and PROMIS PF, UE, and depression did not significantly improve. PROMIS PI significantly improved among simple posterolateral elbow dislocation patients at both six-week and six-month follow-up points (p<0.05). PROMIS PF, UE, and depression did not significantly differ between the time of injury, six-week, or six-month follow-up points. This insight provides a framework for discussion about the utility of PROMIS in evaluating outcomes of elbow dislocations despite the success of PROMIS with other conditions. The results from this study suggest that PROMIS PF and UE may not be adequate in evaluating recovery following elbow dislocations.
